# Hydatid disease of the interventricular septum: Echocardiographic and computed tomography findings

**DOI:** 10.4102/sajr.v24i1.1986

**Published:** 2020-12-15

**Authors:** Brett Wegner, Ruchika Meel, Tamarin Nell, Lamla Nqwata, Michelle Wong

**Affiliations:** 1 Department of Diagnostic Radiology, Faculty of Health Sciences, University of the Witwatersrand, Johannesburg, South Africa; 2 Division of Cardiology, Faculty of Health Sciences, University of the Witwatersrand, Johannesburg, South Africa; 3 Division of Cardiology, Faculty of Health Sciences, Chris Hani Baragwanath Academic Hospital, Johannesburg, South Africa; 4 Department of Diagnostic Radiology, Chris Hani Baragwanath Academic Hospital, Johannesburg, South Africa; 5 Division of Pulmonology, Department of Medicine, Chris Hani Baragwanath Academic Hospital, Johannesburg, South Africa; 6 Department of Medicine, Faculty of Health Sciences, University of the Witwatersrand, Johannesburg, South Africa

**Keywords:** cardiac hydatid disease, interventricular septum, transoesophageal echocardiography, computer tomography, sub-Saharan Africa

## Abstract

Hydatid disease (HD) is prevalent in South Africa, with cardiac HD being a rare but important manifestation to recognise and diagnose. An incidental finding on computed tomography (CT) of the chest in a patient with pulmonary HD prompted further multimodality imaging, which confirmed the presence of cardiac HD involving the interventricular septum. This case report focuses on imaging findings related to cardiac HD, as demonstrated by the CT of the chest and two- and three-dimensional transoesophageal echocardiography. Multimodality imaging is essential to assist in making a diagnosis and providing a detailed assessment of patients with cardiac HD.

## Introduction

Hydatid disease (HD) is a parasitic infestation caused by the *Echinococcus* species. It is endemic in areas of the world where livestock is raised, which include Europe, Middle East, South America, Australasia and sub-Saharan Africa.^1^ The disease is reported to affect men and women equally, with the average age reported to be 32 years.^2^ Human HD commonly affects the liver (50% – 70%) and lungs (20% – 30%).^3^ Cardiac HD is a rare manifestation of human HD and accounts for 0.5% – 2% of all hydatid cases.^4^

This case report presents a rare case of cardiac HD and the imaging findings demonstrated on computed tomography (CT) of the chest and three-dimensional (3D) transoesophageal echocardiography (TOE) imaging.

## Patient presentation

A 43-year-old woman with no comorbidities presented to an academic hospital in Cape Town in 2010 with respiratory symptoms. She previously resided in a rural area of South Africa. In Cape Town, she was diagnosed with pulmonary HD and received medical management with albendazole for 1 year with resolution of her symptoms. From 2010 to 2017, she was lost to follow-up, but was reportedly symptom free.

In 2018, she presented to Chris Hani Baragwanath Academic Hospital (CHBAH) with acute onset respiratory symptoms. Laboratory investigation and imaging suggested HD. Albendazole was commenced with rapid resolution of her symptoms. A chest CT confirmed bilateral pulmonary HD in addition to a hypodense mass located in the interventricular septum (IVS), resulting in partial effacement of the right ventricle. A provisional diagnosis of ventricular thrombus and further investigation with echocardiography was recommended. However, the patient was lost to follow-up for the next 2 years.

**FIGURE 1:**
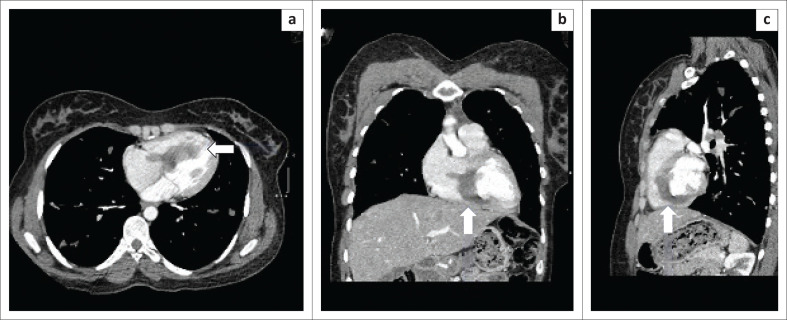
Computed tomography of the chest: (a) axial; (b) coronal; (c) sagittal demonstrating a well-defined interventricular hypodensity protruding into the right ventricle and right atrium (arrows).

**FIGURE 2:**
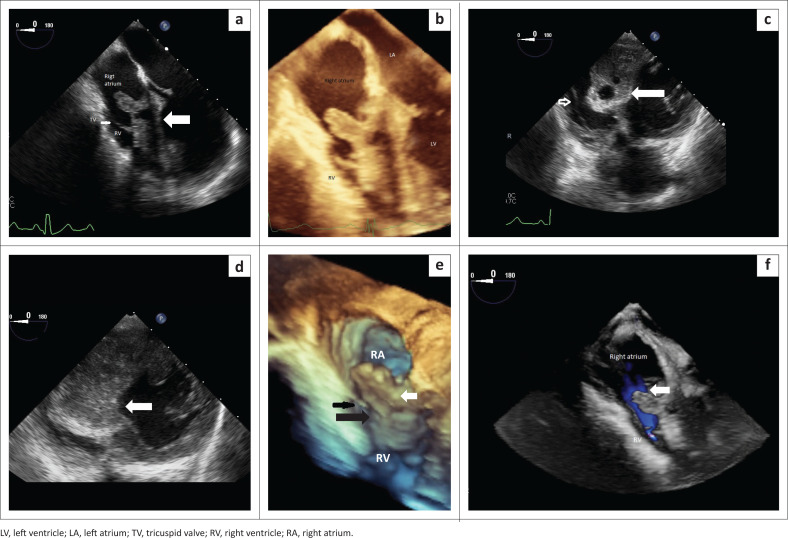
(a) Two- and three-dimensional transoesophageal echocardiography view of the heart at the mid-oesophageal level showing infiltration of the entire interventricular septum (open white arrow) by the hydatid cyst and reduction of the right ventricle to a slit-like cavity. (b) The hydatid cyst protrudes into the right atrium without causing tricuspid valve dysfunction. (c and d) Two-dimensional transoesophageal echocardiography trans-gastric view showing infiltration of the interventricular septum by the hydatid cyst (solid white arrow) and its relation to the tricuspid valve (open arrow, c). Deep trans-gastric view depicting septal hypertrophy due to hydatid disease (white arrow) mimicking asymmetrical septal hypertrophic cardiomyopathy (d). (e and f) Three-dimensional echocardiographic reconstruction of the right atrium (RA) and right ventricle (RV) showing the anterior location of the hydatid cyst protruding into the right atrium (white arrow) relative to the tricuspid valve (black arrow) at the base of the heart (e), and unobstructed blood flow through the tricuspid valve (white arrow) (f).

In May 2020, she again presented to CHBAH complaining of respiratory symptoms. She denied any cardiac symptoms. The general cardiovascular examinations were normal. Laboratory investigations showed normal cardiac enzymes, liver and renal function, and no evidence of sepsis. The *Echinococcus* indirect haemagglutination test was positive (titre of 1:500). A 12-lead electrocardiogram (ECG) was normal. The chest radiograph demonstrated findings consistent with pulmonary HD, which was confirmed at chest CT. In addition to the pulmonary HD findings, the chest CT also demonstrated a hypodensity within the IVS, resulting in partial effacement of the right ventricle and a finger-like protrusion of the hydatid cyst into the right atrium (Figure 1).^5^ Further imaging with 3D TOE confirmed an infiltrating cystic lesion involving the IVS, resulting in expansion of the IVS towards the right ventricle with partial effacement of the right ventricle into a slit-like cavity and protrusion of the cystic lesion into the right atrium without tricuspid valve dysfunction. The cyst demonstrated mixed echogenicity and calcified components (Figure 2).^6^

Imaging techniques used were as follows:

- Contrast-enhanced multi-detector computed tomography (MDCT) was performed on a Canon Aquilion Prime SP (Canon Medical Systems): helical scan mode, 0.35s gantry rotation, collimation 0.5 mm × 80, Sure Exposure TM automatic exposure control, KVP 120, mAs 40–90.
- Philips Epiq 7C, TOE probe 5–7 MHz X7-2t (Philips Healthcare, USA): 2D and 3D TOE.

## Management and outcomes

The patient is currently being managed by a multidisciplinary team (pulmonologist, cardiologist and radiologist). The patient was deemed inoperable because of the extent of the HD. In addition, attempted surgical removal of the cardiac hydatid cysts within the IVS would be technically challenging and would carry a high risk of morbidity and mortality. Because the patient has not had any cardiac complications for at least 2 years, there is no indication for surgical intervention. The patient is receiving medical therapy and is being followed up by specialists of the pulmonology and cardiology services. She remains asymptomatic.

### Ethical consideration

Ethical approval was granted by the University of Witwatersrand Human Research Ethics Committee (HREC) on 26 August 2020 (Reference number: R14/49). Consent was obtained from the patient for the purpose of this case report.

## Discussion

Cardiac HD is a rare manifestation of human hydatidosis as the heart boasts natural resistance to parasitic invasion given the mechanical action of the myocardium.^5^

Humans are considered accidental hosts in the complex life cycle of *Echinococcus* species. Humans acquire the disease after ingestion of parasitic ova found in contaminated food sources.^6^ The parasitic ova hatch in the intestines with subsequent invasion of the mucosa, entering the portal and later the systemic circulation, with the potential to infiltrate any soft tissue within the human body.^7^ *Echinococcus* species gain access to the heart via the coronary circulation and lymphatic networks.^8^ The left ventricle is involved in 60% of cardiac hydatid cases; right ventricle, 10%; IVS, 9%; pericardium, 7%, and left atrial appendage, 6%.^5^

The growth and development of hydatid cysts in the myocardial tissue is slow, and as a result, patients are often asymptomatic.^8^ Symptoms are related to the growth, invasion and size of the hydatid cysts within the myocardial tissue.^5^ Cardiac symptoms range from space-occupying effects causing low cardiac output states, heart failure or valvular dysfunction.^9^ Invasion and irritation of the conduction pathways lead to dysrhythmias, palpitations, syncope or sudden death.^9^ Hydatid cysts can rupture causing pericardial tamponade, pericarditis or systemic dissemination of antigenic material with life-threatening anaphylaxis.^2,9^

Clinical diagnosis of cardiac HD is often difficult as patients may present with no cardiac symptoms, a normal ECG and a normal chest radiograph,^7^ as is the case with this patient. Serological markers can assist with making a diagnosis with a reported sensitivity for hydatid disease in the range of 20% – 60%.^3^

Advanced imaging assists in making the diagnosis of cardiac HD and is mostly reliant on TOE and magnetic resonance imaging (MRI), and to a lesser extent on CT.^4,10^ This is evident in this case with CT identifying an abnormal cardiac lesion with a list of differential diagnoses, including infiltrative cardiomyopathy, hypertrophic cardiomyopathy, tumours and HD of the heart. Imaging with TOE narrowed the differential diagnosis, and in combination with a history of confirmed pulmonary HD and positive serology, cardiac HD was considered the most likely diagnosis. Tissue biopsy and polymerase chain reaction assay identify nucleic acids specific to the *Echinococcus* species; these are regarded as the confirmatory tests for the diagnosis of HD.^11^ A tissue biopsy was not carried out for this patient because of the potential adverse outcome in the setting where tissue confirmation of the diagnosis was considered unnecessary, given the high confidence of an accurate diagnosis carried out by the investigating team using the information gathered through history, laboratory investigations and multimodality imaging.

Transoesophageal echocardiography is an excellent diagnostic tool that is accurate, available, cost-effective and safe to use.^10^ Echocardiography technology continues to improve and allows real-time 3D imaging of the heart, demonstrates the characteristics of a lesion and the anatomical location and assesses the haemodynamic function of the heart in a single examination.^2,12^

The appearance of cardiac HD on TOE is variable, with the majority of cases demonstrating solitary, intramyocardial, oval-shaped cystic lesions with well-defined walls and measuring an average of 3 cm – 5 cm in diameter.^10^ Internal septations are characteristic of daughter cysts.^10^ Hydatid cysts are viable if filled with clear fluid, whilst non-viable cysts are calcified.^13^ Both CT and MRI provide information on the extent and anatomical relationship of the cysts within the heart.^4^ Computed tomography imaging commonly demonstrates well-defined, low-attenuation, spherical cystic lesions (Average Hounsfield unit, 0), with smooth thin walls and variable contrast wall enhancement. Cyst wall calcification and daughter cysts are considered specific imaging features for HD.^4^ Magnetic resonance imaging provides detailed information on the anatomical location, tissue planes and internal characteristics of the cysts.^4,8^ The differential diagnosis to consider with CT and TOE imaging includes intraventricular thrombus, intramyocardial cysts, a variety of cardiac tumours and asymmetrical septal hypertrophic cardiomyopathy.^2^

Magnetic resonance imaging and echocardiography are the preferred modalities for follow-up and assessment of treatment outcomes.^10^ The management of patients with cardiac HD is medical, surgical or a combination of both.^2^ Surgical treatment may be preferable because medical treatment alone does not provide assurance against rupture of a hydatid cyst and life-threatening complications that may follow.^9^ The management of cardiac HD is multidisciplinary, with therapeutic decisions based on the characteristics of the cardiac HD, patients’ symptoms and comorbidities, as well as the surgical expertise available at the treating institution.^14^

## Conclusion

This case report highlights that cardiac HD as a rare, but important, manifestation of human hydatidosis that is often silent and overlooked. Patients known to have HD and presenting with cardiac pathology should always be referred for advanced multimodality imaging, with particular reference to TOE in order to appropriately assess for cardiac involvement. Patient-specific treatment is guided by the clinical presentation, multimodality cardiac imaging and a multidisciplinary team.
